# Mother–infant stress contagion? Effects of an acute maternal stressor on maternal caregiving behavior and infant cortisol and crying

**DOI:** 10.1111/jcpp.14119

**Published:** 2025-01-21

**Authors:** Nina Bruinhof, Roseriet Beijers, Hellen Lustermans, Carolina de Weerth

**Affiliations:** ^1^ Department of Cognitive Neuroscience Radboud University Medical Center, Donders Institute for Brain, Cognition and Behaviour Nijmegen The Netherlands; ^2^ Department of Social Development, Behavioural Science Institute Radboud University Nijmegen The Netherlands

**Keywords:** Postnatal stress, TSST, HPA‐axis, stress contagion, caregiving

## Abstract

**Background:**

Postpartum maternal distress has been associated with adverse infant outcomes. A potential pathway of how maternal distress affects infant outcomes could be alterations in maternal caregiving behavior. However, the associations between maternal distress, caregiving behavior, and infant outcomes have never been tested in a controlled experiment. This preregistered study utilized an experimental design to investigate the effects of an acute maternal stressor on infant cortisol and crying and the possible mediating role of maternal caregiving behavior.

**Methods:**

Mother‐infant dyads (*N* = 91) participated in a lab visit at 8 weeks postpartum, where mothers were separated from their infants to either perform a Trier Social Stress Test (TSST) or a control task. The task was immediately followed by a mother‐infant interaction to assess maternal caregiving behavior and infant cortisol and crying.

**Results:**

Our structural equation model found no differences between conditions (stressor/control) on maternal caregiving behavior and infant response to maternal stress. Secondary findings revealed that higher quality of maternal caregiving behavior was related to lower levels of infant crying and lower cortisol levels at the end of the visit, but not cortisol at reunion.

**Conclusions:**

Our findings do not support the occurrence of mother‐infant stress contagion in this experimental setting but do indicate a link between maternal caregiving behavior and infant behavioral and cortisol responses. Given the high prevalence of maternal mental health problems and their possible negative association with offspring development, further (experimental) research is needed to understand just how maternal postpartum distress affects young infants.

## Introduction

The transition to parenthood is often accompanied by maternal psychological distress (i.e., symptoms of depression, anxiety, and stress) (Dipietro, Costigan, & Sipsma, 2008; Monk et al., 2022). During pregnancy, increased maternal distress predicts poorer infant outcomes, including compromised neurodevelopment and increased negative affect, via various physiological mechanisms (Beijers, Buitelaar, & de Weerth, 2014; Van den Bergh et al., 2020). Following childbirth, maternal psychological distress continues to be associated with compromised infant outcomes (Bates, Militello, Barker, Villasanti, & Schmeer, 2022; Field, 2018; Kingston, Tough, & Whitfield, 2012). During the postpartum period, one mechanism through which maternal psychological distress might impact the infant is through the transmission of distress, also referred to as stress contagion. During this process of stress contagion, negative affective states are transferred from one partner to another (Engert, Linz, & Grant, 2019). When a mother is stressed, the infant might pick up on this stress and respond with their own cortisol or behavioral stress response. Only a few studies have investigated causal effects of maternal exposure to a stressor on infant outcomes. Waters and colleagues (2014; 2017), investigated potential stress contagion by exposing the mother to a laboratory stressor and then reuniting her with her 12‐ to 14‐month‐old infant. Maternal exposure to a stressor resulted in more sympathetic activation and avoidance behavior in the infant after reunion, as compared to infants of control group mothers. Whether the same would be true in younger infants is yet unknown. Therefore, our first goal was to investigate whether in the first postnatal months, maternal exposure to a stressor subsequently leads to an infant cortisol and behavioral response. The relevance of this goal is given by this postnatal period being critical due to rapid infant brain development, hypothalamus–pituitary–adrenal (HPA) axis maturation, and the early stages of the development of the mother‐infant attachment bond (Evans & Porter, 2009; Hodel, 2018). Furthermore, a better causal understanding of stress transmission from mother to young infant is important for designing effective (preventive) interventions (Perlman, Lunkenheimer, Panlilio, & Pérez‐Edgar, 2022).

A potential pathway for how maternal distress can affect infant outcomes is through alterations in her caregiving behavior (Choe, Olson, & Sameroff, 2013). Maternal caregiving behavior often decreases in quality when struggling with postpartum distress (Booth, Macdonald, & Youssef, 2018). Moreover, low quality of maternal caregiving behavior has been associated with adverse infant outcomes (Berry, Blair, Willoughby, Granger, & Mills‐Koonce, 2017; Deans, 2020) and found to mediate the association of maternal distress to infant behavior (Choe et al., 2013). However, this relation has never been tested in a controlled experiment. Our second research goal was to investigate whether maternal exposure to a stressor affects the quality of her caregiving behavior, which in turn mediates the association between maternal stressor exposure and infant cortisol and behavioral response.

This experimental study was carried out in a low‐risk, healthy sample of mothers and their 8‐week‐old infants. Mothers were assigned to a stressor (i.e., Trier Social Stress Test; Kirschbaum, Pirke, & Hellhammer, 1993) or control condition. Our main hypotheses were: (a) after reunion, infants of mothers exposed to the stressor will have higher cortisol and crying responses compared to infants from mothers in the control condition, and (b) this association will be (partially) mediated by lower maternal caregiving quality. Secondary hypotheses were, first, because individuals already experiencing daily distress are more susceptible to the effects of the acute stressor (Allen, Kennedy, Cryan, Dinan, & Clarke, 2014; Chida & Hamer, 2008), and many mothers experience distress postpartum (Browne, Bossenbroek, Kluft, van Tetering, & de Weerth, 2020): (c) the effects of maternal exposure to the stressor on caregiving quality and infant cortisol and crying will be stronger for mothers with pre‐existing high levels of postpartum distress. It is important to note that we refer to these general levels of postpartum distress as measured by questionnaires as “distress”. Moreover, we refer to the experimental manipulation as a “stressor” and to the maternal response to this stressor with “stress”. Second, as separation from the newborn might be stressful for all postpartum mothers, regardless of condition, we hypothesized that (d) for the group as a whole, mothers' heightened cortisol concentrations and negative affect will be associated with higher levels of infant cortisol and crying, and this association will be (partially) mediated by lower caregiving quality.

## Methods

### Participants

**Figure 1:**
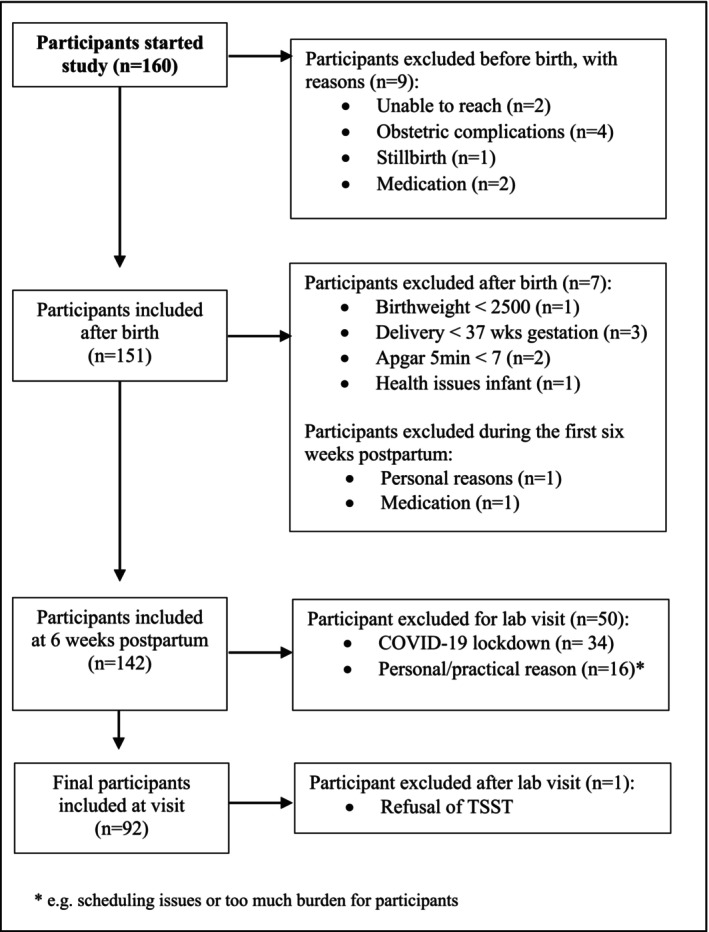
Participant flowchart

The current study is a randomized controlled experiment within the ongoing longitudinal SMILEY study (Study of Microbiota and Lifestyle in the Early Years) that follows a low‐risk community sample of 160 healthy women and their infants from 18 weeks of pregnancy. Participants were recruited through our Baby and Child participant database (https://babyandchild.nl/en/), midwifery practices in surrounding areas, and social media. Maternal inclusion criteria were: singleton pregnancy, mastery of the Dutch language, ≥18 years of age, no severe physical/mental health issues, <21 weeks of gestation at enrollment, and pre‐pregnancy BMI ≤30. Infant inclusion criteria were: born at ≥37 weeks of pregnancy, birth weight ≥2,500 g, 5‐min Apgar score ≥7, and absence of serious malformations and diseases. An overview of excluded participants is presented in the participants flowchart (Figure 1). In total, 142 mother‐infant dyads were participating at 8 weeks postpartum, of which 92 dyads visited the lab between December 2020 and January 2022. Participants gave informed consent. From inclusion until 12 weeks postpartum, they received three small gifts and 100 euros. The hypotheses and statistical analyses of the study were preregistered on the Open Science Framework (https://doi.org/10.17605/OSF.IO/N5VPS). The study procedures were approved by the Ethics Committee Faculty of Social Sciences of the Radboud University (SW2017‐1303‐497), including three amendments (ECSW‐2019‐051, ECSW‐2019‐106, ECSW‐2020‐021).

### Procedure

**Figure 2:**
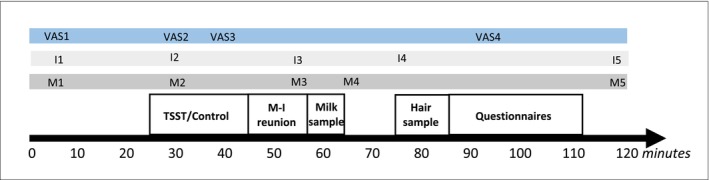
Timeline laboratory visit. VAS1–VAS4: Visual Analogue Scales. M1–M5: maternal saliva samples 1–5, I1‐I5: infant saliva samples 1–5. TSST: Trier Social Stress Test. M‐I reunion: mother‐infant reunion. This timeline shows when the maternal and infant cortisol samples were collected. The timeline reflects the study protocol without interruptions (i.e., due to infant feeding or diaper change); however, adaptations were made to meet the infant's needs (e.g., infant feeding prior to manipulation or after sampling milk). Preregistered rules determined if deviations of the protocol were acceptable (see Table 1 for rules). Note that the hair sample, milk sample, and questionnaires were not part of the current study

Mothers and their infants were invited to visit the lab around 8 weeks postpartum (*M* = 8.21, *SD* = 1.33). Mothers filled in questionnaires at home on general distress at 2 and 6 weeks postpartum. Upon arrival at the lab, mothers first received instructions. Then mothers were separated from their infants. Subsequently, the manipulation (stressor or control task) for the mother followed. In the meantime, a babysitter (i.e., a research assistant or a partner, parent, or friend of the mother) took care of the infant in a different room. After, mother and infant were reunited, and a mother‐infant interaction took place with the aim of observing maternal caregiving quality and infant behaviors. The infant was thus not directly exposed to the stressor or control task the mother performed. In total, the lab visit lasted around 2 hr (see Figure 2). Throughout the lab visit, five saliva samples of both mother and infant were collected to measure cortisol concentrations (see Table 1). Visits were scheduled in the afternoon (between 12:00 and 17:30 h) to minimize the effect of natural cortisol circadian rhythm on cortisol reactivity. Mothers were instructed not to eat or drink anything other than water or caffeine‐free tea and to refrain from physical activity for at least 1 hr before the visit.

**Table 1:** Saliva cortisol sample information

Sample	Reflecting moment	Corresponding mother's VAS	Removing when?	*N* samples removed
*Mother*				
M1	Baseline^a^	N/A	N/A	N/A
M2	Start lab visit	VAS1	<35 min after VAS1	2
M3	Preparation manipulation	VAS2	>35 min after start manipulation	0
M4	Waiting after manipulation	VAS3	>35 min after end manipulation	6
M5	End of visit	VAS4	<45 min after end manipulation	5
*Infant*				
I1	Baseline^a^	N/A	N/A	N/A
I2	Start lab visit	N/A	<35 min after VAS1	2
I3	Babysitting	N/A	>35 min after start manipulation	0
I4	Reunion^b^	N/A	>30 min after end mother‐infant interaction	0
I5	End of visit	N/A	<45 min after end manipulation	0

M1–M5 is mother sample 1–5, I1 to I5 is infant sample 1–5, VAS, Visual Analogue Scale; N/A, not applicable; min = minutes.

^a^ Reflects around 20 min before the visit, mostly when the mothers were traveling to the visit.

^b^ Proposed contagion moment between mother and infant.

#### Randomization

In pregnancy, participants were randomized into two conditions: a stressor condition (i.e., Trier Social Stress Test (TSST) and a control condition). The randomization was stratified using a random block randomization method to ensure a comparable distribution of primi‐ and multiparous women in both conditions. With a computer‐generated allocation sequence, mothers were randomly assigned to either the TSST or control condition (1:1) in blocks of 4 or 6. An independent researcher, who was not involved in the study, prepared the schedule and concealed the allocation sequence in stapled envelopes. Before the prenatal visit, the main researcher opened the envelopes to allocate participants to the stressor or control condition. The mothers that were randomly assigned to the TSST condition in pregnancy did the control condition postpartum and vice versa. The pregnancy visit studied effects of the acute stressor on dietary intake (see Lustermans, Beijers, Vis, Aarts, & de Weerth, 2024). Participants were blinded for group allocation and unaware of the different conditions until debriefing at 12 weeks postpartum.

#### Manipulation

In the stressor condition, participants performed the Trier Social Stress Test (TSST; Kirschbaum et al., 1993). Social evaluative lab stressors, including the TSST, have been shown to provoke a cortisol and subjective stress response in most participants and can be seen as the golden standard for eliciting acute stress (Zänkert, Bellingrath, Wüst, & Kudielka, 2019). Due to the ongoing COVID‐19 pandemic, the jury was online through a live connection (Lustermans et al., 2024). The control condition (i.e., separation‐only) participants performed a fabric feeling task (Van Strien, Roelofs, & De Weerth, 2013).

For both conditions, the manipulation consisted of three elements: a preparation phase (5 min), a manipulation phase (10 min), and a waiting phase (5 min). For the stressor condition, the researcher explained the task according to the TSST protocol (Kirschbaum et al., 1993). The mothers had 5 min to prepare for a presentation about their dream job, after which they were brought to a different room for their presentation. On a TV screen, two female judges were present wearing white lab coats. The judges were trained to look strict, give no positive feedback, and take notes during the assignment. The participants performed a presentation (5 min) and an arithmetic task, in which participants had to count back from 1,687 in increments of 13 (5 min). They were recorded by a visible camera. After the arithmetic task, the researcher came back to the room, instructing the participant to wait for a few more minutes for the jury to evaluate the performance. After 5 min of waiting, the participant was told she did well on the task and brought back to be reunited with her infant.

For the control condition, the setup mimicked the TSST set‐up. Mothers were first instructed to read paper instructions about a fabric‐feeling task for 5 min. Afterward, they were brought to a different room (i.e., the same room as the TSST, however, without camera or jury) to rate different fabrics (e.g., denim, lace, satin) on different characteristics (e.g., softness, pleasantness, warmth). After 10 min, the mother was instructed by the researcher to stop the task and wait for the researcher to come back. After 5 min of waiting, she was reunited with her infant.

#### Mother–infant interaction

After mothers had been reunited with their infants, a mother‐infant interaction immediately followed with the aim of observing maternal caregiving quality and infant behaviors. To increase variability between mothers and ecological validity, mothers were instructed to undress, weigh, and then dress their infants again. During this interaction, mothers and their infants were alone in the room and filmed by three corner cameras. The duration of the mother‐infant interaction was 13 min, regardless of how far mothers were with the instructed tasks. This duration was chosen to ensure the interaction was long enough to reliably observe maternal caregiving behaviors.

### Measures

#### Maternal response to acute stressor

##### Maternal cortisol reactivity

During the lab visit, mothers collected five saliva samples (M1–M5) through passive drooling through a straw. Times of collection were dependent on the manipulation (see Table 1). Participants were instructed to collect at least 0.5 ml of saliva. After the visit, samples were stored in a freezer at −20°C. Cortisol concentrations were determined at the Laboratory of Endocrinology at UMC Utrecht. They were measured without extraction using an in‐house competitive radio‐immunoassay employing a polyclonal anticortisol‐antibody (K7348). [1,2‐3H(N)]‐Hydrocortisone (PerkinElmer NET396250UC) was used as a tracer. The lower limit of detection was 1.0 nmol/L. We calculated maternal reactivity as the maximum cortisol increase. We first selected the lowest baseline (either sample M1 or M2) and the highest peak (either sample M3 or M4) and used the formula: highest peak – lowest baseline. This method of direct comparisons between defined time points was chosen to capture the acute effects of the stressor more precisely and in accordance with previous papers to assess the individual variation in reactivity (de Veld, Riksen‐Walraven, & de Weerth, 2012; Simons, Zijlmans, Cillessen, & De Weerth, 2019). We also conducted sensitivity analyses for cortisol reactivity by testing the peaks M3 and M4 separately, subtracting each from the lowest baseline (M1 and M2).

##### Maternal negative affect reactivity

To assess maternal negative affect in response to the stressor, mothers filled in the Dutch translation of the Visual Analogue Scales (VAS; Monk, 1989) at four time points (VAS1 to VAS4). The times of filling in the VAS reflected the timing of the saliva samples and reactivity to the stressor (see Table 1). The VAS consisted of 4 items asking about feelings of tenseness, calmness, happiness, and sadness. Mothers filled in paper versions of the VAS by setting a cross on lines ranging from “not at all” on the left to “a lot” on the right. Scores were calculated by measuring the distance from the left of the line to the cross in millimeters (range 0–117). Total scores were calculated following the formula for global affect = [(happy) + (calm) + 200‐(sad)‐(tense)]/4 (see Monk, 1989). Total scores were reversed so that higher scores indicated more negative affect. To assess maternal negative affect reactivity, we followed a similar procedure as for cortisol reactivity by subtracting the highest peak (either VAS2 or VAS3) from baseline (VAS1). Similarly to cortisol reactivity, we also ran sensitivity analyses with VAS2 and VAS3 as peaks separately.

#### Infant response to the mother's stressor

##### Infant salivary cortisol concentrations

To assess infant cortisol concentrations, five saliva samples (I1–I5) were taken. Saliva of the infant was collected either by the mother or the researcher by gently swabbing the infant's mouth with absorbent eye sponges for approximately 1 min (de Weerth, Jansen, Vos, Maitimu, & Lentjes, 2007). The timing of the infant cortisol samples corresponds to the timing of the mother samples, except for sample I4, which for the infant reflected the reunion (see Table 1). To avoid contamination of saliva samples by milk, the lab protocol prescribed that infant cortisol was not assessed immediately after feeding. After collection, the samples were centrifuged and stored at −20°C. The laboratory analysis of the infant samples was identical to the maternal samples. Only samples I4 and I5 reflected moments after the manipulation (i.e., I4: reunion and I5: end of the visit) and therefore were expected to differ between the stressor and control groups.

##### Infant crying

Infant crying was scored using the 13‐min videos of the mother‐infant interaction. Every interval of 15 s was scored by the first author and a research assistant as containing infant crying or not (yes = 1, no = 0). Crying was defined as sounds with clear negative affect. Because videos differed slightly in duration, for every video, a percentage was calculated representing the proportion of intervals with infant crying. Thirty percent of the videos were double scored, and agreement between observers was good (92.99%). Observers of the videos were blind to the mother's condition (stressor/control) and other study variables (i.e., maternal postpartum distress levels).

#### Maternal caregiving behavior

Quality of maternal caregiving behavior was scored using the videos of the 13‐min mother‐infant interactions. Mothers were rated on four different caregiving behavior scales: sensitivity, cooperation, positive regard for the child, and negative regard for the child. High‐quality caregiving behavior is referred to as sensitive (i.e., the caregiver responds timely and adequately to the infant's needs and signals) and cooperative (i.e., the caregiver adjusts behavior to the infant and does not interfere with the infant's ongoing activity; Ainsworth, Blehar, Waters, & Wall, 1978). Moreover, caregiving behavior quality often includes positive and negative regard to the child, with more positive and less negative regard promoting infant development (Brooker, Davidson, & Goldsmith, 2016; Sheinkopf et al., 2017). Maternal sensitivity and cooperation were assessed using the Ainsworth scales (Ainsworth et al., 1978) with a 9‐point scale ranging for sensitivity from 1 (highly insensitive) to 9 (highly sensitive) and for cooperation from 1 (highly interfering) to 9 (conspicuously cooperative). Positive and negative regard towards the child were scored on 7‐point scales and were assessed using the Observational Record of the Caregiving Environment (ORCE) scales (NICHD Early Child Care Research Network, 1996). Higher scores indicated more positive and more negative regard towards the child, respectively. The four caregiving measures were added into the models as a latent factor. Videos were scored by the first author and two trained assistants, with 30% of the videos being double scored. Observers of maternal caregiving behavior were also blind to the mother's condition (stressor/control) and other study variables (i.e., maternal postpartum distress levels). Interrater agreement (Weighted Cohen's kappa, *k*) on the measures was excellent: sensitivity (*k =* .90), cooperation (*k =* .86), positive regard (*k =* .90), and negative regard (*k =* .93). All measures were significantly correlated with each other, with correlations ranging from −.35 to .92.

#### General maternal postpartum distress

General maternal postpartum distress consisted of postpartum depressive, anxiety, and stress symptoms at 2 and 6 weeks postpartum. The questionnaires used were the Edinburgh Postnatal Depression Scale (EPDS; Cox, Holden, & Sagovsky, 1987), the State subscale of the State Trait Anxiety Inventory (STAI; Spielberger, 1985), and the Perceived Stress Scale‐10 (PSS‐10) questionnaire (Cohen et al., 1983). The EPDS is a widely used questionnaire for perinatal depression. The questionnaire consists of 10 items, with a 4‐point scale ranging from 0 to 3. The total scores range from 0 to 30, with higher scores indicating higher levels of depressive feelings. A score of 11 is advised as a cutoff score for an indication of subclinical depression (Levis, Negeri, Sun, Benedetti, & Thombs, 2020). The STAI State consists of 20 items on a 4‐point scale ranging from 1 to 4 and is often used to measure anxiety. A total score ranges from 20 to 80, with higher scores indicating higher‐reported levels. A score >40 is commonly used as a cutoff score for subclinical anxiety (Dennis, Coghlan, & Vigod, 2013). Lastly, the PSS‐10 is a questionnaire to measure stress. The questionnaire consists of 10 items with a 5‐point scale, ranging from 0 and 4. Total scores range from 0 to 40, with higher scores indicating higher reported levels. While the EPDS assesses feelings of the past week and the STAI feelings of that moment, the PSS‐10 scores indicate stress of the past month; therefore, we only used the PSS‐10 assessed at 6 weeks postpartum. Internal consistency for the questionnaires in the current study was excellent: EPDS (2 weeks ωt = .91, 6 weeks ωt = .86), STAI (2 weeks ωt = .94, 6 weeks ωt = .93), and PSS‐10 (ωt = .91). The questionnaires were standardized and summed to a composite score called general maternal postpartum distress.

#### Covariates

No covariates were added to the analyses answering hypotheses 1 to 3. As the participants were randomized, groups are assumed not to differ systematically on variables that may confound the results (de Boer, Waterlander, Kuijper, Steenhuis, & Twisk, 2015). For hypothesis 4, covariates are included. The selection of covariates was made with the use of a directed acyclic graph (DAG) following Cinelli, Forney, and Pearl (2024). The covariates that subsequently were included for hypothesis 4 were: familiarity with the babysitter during separation (i.e., brought by mother vs. researcher), infant cortisol at baseline (I3), breastfeeding before manipulation (yes/no), infant age (in days), and maternal food intake less than 1 hr before the visit (yes/no).

### Missing data

One participant was excluded from the 92 lab visits due to refusing to perform the TSST. The final sample consisted of 91 participants, with 47 in the stressor condition and 44 in the control condition. Women who withdrew from the postnatal phase of the study did not significantly differ from those who did visit the lab on the following variables: maternal depressive, anxiety, and stress symptoms at 2 or 6 weeks postpartum (all *p*'s > .30), maternal education (*p* = .09), and parity (*p* = .22).

For the maternal cortisol samples, total missingness was 2.20%, and for infant cortisol samples, total missingness was 24.84%. Missingness for infant samples was higher than for maternal samples, mostly due to an insufficient volume of infant saliva or inability or refusal to collect when the infant was crying or sleeping. Sample 1 was on average taken 7 min after arrival (SD = 4, range = 0–33). Samples 2–5 were screened on sample time and removed when taken too late (see Table 1). Missingness of infant cortisol samples did not depend on postnatal depressive, anxiety, and stress symptoms at 2 and 6 weeks (all *p*'s > .36). Furthermore, missingness was also not related to maternal education (*p* = .11) nor parity (*p* = .27). We did find that infants with a missing cortisol sample at reunion cried more at that same moment (*p* < .01), and older infants had higher rates of missing data (*p* < .001). Our models corrected for missing values by means of imputation techniques (see Statistical analyses section).

### Statistical analyses

Before analyzing the data, cortisol values were checked for implausibly high cortisol concentrations higher than 60 nmol/L (Miller, Plessow, Rauh, Gröschl, & Kirschbaum, 2013). No implausible cortisol values for either mother or infant were detected in our data. Then, all study variables were checked for outliers (greater or smaller than 3 *SD* from the mean). The maternal cortisol and negative affect reactivity were tested for outliers per group (stressor/control). For the stressor group we had an outlier for M3 (*n* = 1). For the control group we had outliers for M1 (*n* = 1), M2 (*n* = 1), M3 (*n* = 1), and VAS3 (*n* = 1). For the whole group we had the following outliers: I5 (*n* = 1), crying ratio (*n* = 1), I1 (*n* = 2), I3 (*n* = 2), I4 (*n* = 2), maternal negative regard (*n* = 3), maternal depressive symptoms (*n* = 1), and anxiety symptoms (*n* = 1). Outliers were winsorized, meaning the value was brought back to 3 *SD* from the mean. Variables were normally distributed except M4, I3, I4, I5, infant crying, maternal depressive symptoms, and anxiety symptom scores. These variables were log‐transformed, except for the maternal depressive symptoms score, which was square root transformed due to containing 0 values. As a manipulation check, we compared maternal cortisol and negative affect reactivity between the conditions (stressor/control) with a MANOVA.

For our main analyses we used structural equation modeling (SEM). To refrain from multiple testing and to be parsimonious, we used as few statistical tests as possible. Therefore, we used SEM to simultaneously test Hypothesis 1 and 2 (i.e., maternal condition (stressor/control) on infant response, mediated by caregiving quality). We performed three SEMs, one for each different outcome (i.e., infant cortisol at reunion, infant cortisol at the end of the visit, and infant crying), with the two different conditions dummy coded as predictors (stressor/control; see Figure S1A).

To investigate hypothesis 3, whether mothers with higher general postpartum distress showed a stronger association between stressor exposure and infant outcomes, we performed four regression models with maternal general postpartum distress as the moderator, the two different conditions (stressor/control) as predictor, and (a) infant cortisol at reunion, (b) infant cortisol at the end of the visit, (c) infant crying, and (d) maternal caregiving behavior as outcomes. Due to the regression analyses, maternal caregiving behavior was added as a standardized sum score, instead of the latent factors used in SEM.

For secondary hypothesis 4, whether caregiving behavior mediated the association between maternal reactivity and infant response, we performed six SEMs with the continuous predictor: (1) maternal cortisol reactivity or (2) maternal negative affect reactivity, and with the infant outcomes: (a) cortisol at reunion, (b) cortisol at the end of the visit, (c) crying, and the mediator maternal caregiving behavior. We added the previously mentioned covariates (babysitter during separation, infant cortisol at baseline, breastfeeding before, infant age, and maternal food intake less than 1 hr before the visit) to all these models (see Figure S1B).

All analyses were performed in R. All SEMs displayed a negative variance for maternal sensitivity in the latent factor caregiving; therefore, maternal sensitivity within the latent factor was set to 0. For the mediation analyses, we used lavaan (Rosseel, 2012) for a classic mediation set‐up with the direct effect of X on Y and the indirect effect of X on Y via M. Missing data was imputed in the model with Full Information Maximum Likelihood (FIML) for the SEMs and Multiple Imputation by Chained Equations (mice; van Buuren & Groothuis‐Oudshoorn, 2011) for the regression analyses.

## Results

### Descriptive analyses

Table 2 presents the descriptive statistics, and Table 3 presents the correlations between the study variables. More infant crying was correlated to higher infant cortisol concentration at reunion (I4) and at the end of the visit (I5). Moreover, higher levels of general postpartum distress were correlated to more infant crying. Infant cortisol samples 4 and 5 were highly correlated.

**Table 2:** Demographic characteristics

	Total (*N* = 91)^a^		Stressor (*n* = 47)		Control (*n* = 44)	
	*M* (*SD*)/% (*N*)	Range	*M* (*SD*)/% (*N*)	Range	*M* (*SD*)/% (*N*)	Range
Maternal age (in years)	32.56 (3.57)	22.95–42.27	33.22 (3.82)	22.95–38.38	32.54 (3.33)	25.79–42.27
Infant age (in weeks)	8.21 (1.33)	6.29–11.57	8.24 (1.23)	6.29–11.00	8.18 (1.23)	6.29–11.57
Infant sex (% boys)	50.55% (46)		54.19% (25)		47.73% (21)	
Parity (% nulliparous)	48.35% (44)		53.19% (25)		43.18% (19)	
Dutch background	91.21% (83)		89.26% (42)		93.18 (41)	
*Education*						
Low/medium	10.99% (10)		10.64% (5)		11.36% (5)	
High	89.01% (81)		89.36% (42)		88.64% (39)	
*Maternal cortisol*						
Cortisol 1	8.80 (2.84)	2.20–19.60	8.94 (2.50)	2.20–16.40	8.67 (3.17)	3.70–19.60
Cortisol 2	8.16 (2.69)	2.50–18.20	8.55 (2.86)	2.50–14.40	7.77 (2.49)	3.40–18.20
Cortisol 3	8.71 (3.75)	2.30–24.00	9.98 (4.53)	2.30–24.00	7.42 (2.08)	3.10–14.30
Cortisol 4	8.87 (4.39)	2.40–27.00	11.10 (5.35)	2.40–27.00	6.97 (1.91)	3.10–12.30
Cortisol 5	6.12 (2.50)	1.60–13.80	7.01 (2.85)	1.80–13.80	5.20 (1.67)	1.60–8.10
*Maternal negative affect reactivity*						
VAS 1	39.63 (13.42)	16.50–78.50	42.06 (15.08)	16.50–78.50	37.03 (10.97)	17.50–69.00
VAS 2	51.87 (20.74)	16.50–93.75	64.68 (17.94)	17.75–93.75	38.69 (13.60)	16.50–74.63
VAS 3	57.21 (25.31)	17.00–118.75	73.60 (23.46)	20.50–118.75	40.07 (12.71)	17.00–81.25
VAS 4	37.13 (12.76)	16.50–68.50	40.13 (12.33)	16.50–68.50	48.38 (12.80)	16.75–65.13
*Infant cortisol*						
Cortisol 1	10.43 (5.83)	4.30–34.00	10.51 (5.28)	4.50–25.00	10.36 (6.37)	4.30–34.00
Cortisol 2	10.78 (5.02)	2.60–24.00	11.28 (4.91)	2.60–22.00	10.30 (5.15)	4.10–24.00
Cortisol 3	10.49 (5.05)	3.90–28.00	10.73 (4.93)	4.00–26.00	10.23 (5.22)	3.90–28.00
Cortisol 4	12.99 (8.05)	5.50–46.00	13.50 (7.91)	6.20–45.00	12.38 (8.33)	5.50–46.00
Cortisol 5	10.17 (4.27)	4.30–23.00	10.60 (4.22)	5.50–21.00	9.71 (4.35)	4.30–23.00
Infant crying^b^	19.08%	0%–100%	21.13%	0% –86.27%	16.97%	0%–100%
*Maternal caregiving*						
Sensitivity	5.72 (2.16)	1–9	5.55 (2.11)	2–9	5.91 (2.22)	1–9
Cooperation	5.48 (2.25)	1–9	5.36 (2.28)	1–9	5.60 (2.24)	1–9
Positive regard	4.99 (1.26)	2–7	4.86 (1.13)	3–7	5.12 (1.38)	2–7
Negative regard	1.34 (.55)	1–3	1.39 (.58)	1–3	1.30 (.51)	1–3
*General postpartum distress*						
EPDS	5.16 (3.61)		5.99 (4.13)		4.27 (2.73)	
2 weeks postpartum	5.27 (4.30)	0–22	6.26 (5.02)	0–22	4.23 (3.10)	0–11
6 weeks postpartum	5.04 (3.93)	0–16	5.72 (4.30)	0–16	4.32 (3.39)	0–14
STAI State	30.66 (6.35)		31.79 (7.30)		29.47 (4.94)	
2 weeks postpartum	30.76 (7.66)	20–54	32.11 (8.69)	20–54	29.32 (6.19)	20–46
6 weeks postpartum	30.57 (7.07)	20–58	31.47 (7.42)	20–55	29.64 (6.62)	21–58
PSS‐10 6 weeks postpartum	10.96 (5.89)	0–28	11.96 (6.47)	0–28	9.89 (5.05)	2–24
Babysitter (%research assistant)	61.54% (56)		61.70% (29)		61.36% (27)	
Breastfeeding before manipulation (%yes)	36.67% (33)		36.17% (17)		37.21% (16)	

Raw values before winsorizing, before log or square‐root transformation. Education = low/medium: secondary education or vocational education; high: bachelor or master's degree or higher. Cortisol values in nmol/L. *N*, total sample; *M*, mean; *SD*, standard deviation.

^a^ 92 lab visits were performed, but 1 participant was excluded from the analyses (see Methods).

^b^ Percentage of 5‐s intervals when the infant was crying during the mother‐infant interaction, ranging from none (0%) to the whole interaction (100%).

**Table 3:** Pearson Correlation Matrix among all variables in the study (*N* = 91)

	1.	2.	3.	4.	5.	6.	7.	8.
1. Maternal condition^a^	–	.39***	.68***	−.11	.11	.14	.10	.22*
2. Maternal cortisol reactivity		–	.26*	.02	.18	.20	.12	−.02
3. Maternal negative affect reactivity				.02	.02	.08	−.05	.14
4. Maternal total caregiving				–	−.11	−.22	−.32**	−.10
5. Infant cortisol at reunion (I4)					–	.86***	.54***	−.03
6. Infant cortisol at the end of the visit (I5)						–	.36**	.02
7. Infant crying							–	.21*
8. General postpartum distress								–

Correlations after winsorizing and log or square‐root transformations. Maternal caregiving behavior is a sum score of sensitivity, cooperation, positive regard, and negative regard.

^a^ Condition is dummy coded with 0 = control condition and 1 = stressor condition. The correlations with maternal condition were calculated with Point‐Biserial correlations.

***<.001; **<.01; *<.05.

### Preliminary analyses

**Figure 3:**
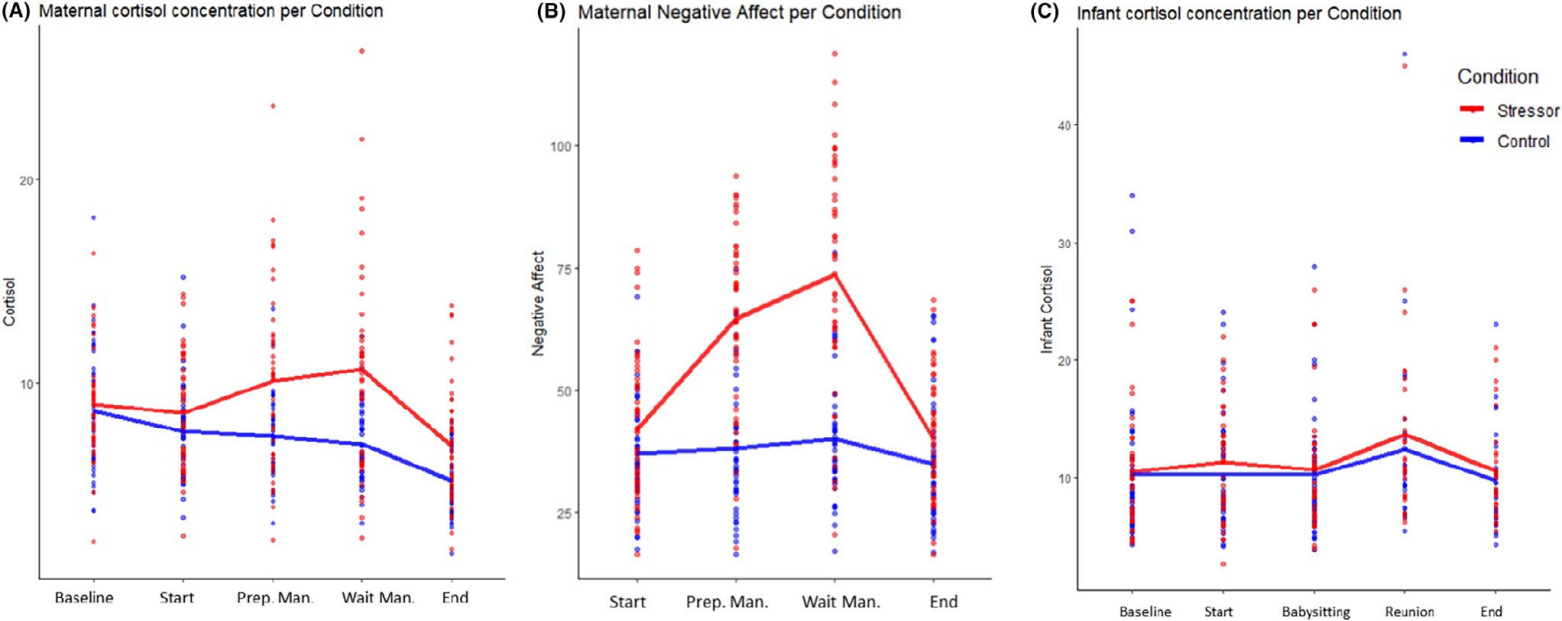
Maternal cortisol concentrations, maternal negative affect, and infant cortisol during the lab visit. Start = start of the lab visit, Prep. Man = preparation manipulation; Wait Man. = waiting after manipulation. Exact timings of assessment can be found in Table 1

We examined maternal reactivity differences between the two experimental conditions (TSST/control; see Figure 3). As expected, a MANOVA revealed that mothers in the stressor condition had higher cortisol levels during the preparation for the manipulation (M3, *p* < .001), waiting after the manipulation (M4, *p* < .001), and at the end of the visit (M5, *p* < .001). Similarly, for negative affect, we found that mothers in the stressor condition had higher levels of negative affect during the preparation for the manipulation (VAS2, *p* < .001) and waiting after the manipulation (VAS3, *p* < .001), but not at the end of the visit (VAS4, *p =* .07). Infant cortisol concentrations at reunion and at the end of the visit did not significantly differ between maternal conditions (stressor/control task; see Figure 3; see Table 3 for correlations).

#### Maternal acute stressor on caregiving behavior quality and infant cortisol and crying

To test our first hypothesis of the effect of the maternal stressor on infant response (i.e., infant cortisol at reunion, infant cortisol at the end of the visit, and infant crying) via maternal caregiving behavior quality, we ran three SEMs (see Table 4). Our results showed no direct effect of maternal exposure to the acute stressor on infant cortisol or crying, nor did maternal caregiving quality mediate the association between the acute stressor and infant response. However, higher maternal caregiving behavior quality was associated with lower levels of infant cortisol at the end of the visit and less infant crying, but not infant cortisol levels at reunion. The fit of the models can be found in Table  S1.

**Table 4:** Results from three mediation models (hypothesis 1 and 2) of condition (stressor/control) on infant response to maternal stress divided into (1) infant cortisol at reunion, (2) infant cortisol at the end of the visit, and (3) infant crying, mediated by caregiving behavior quality

Model description	Estimate	Standardized estimate (β)	*SE*	*p*‐Value	CI	
					LL	UL
*Model 1. Infant cortisol at reunion (I4)*						
Condition → Infant Cortisol I4	.04	.11	.05	.43	−.06	.14
Condition → Caregiving	‐ .39	−.09	.46	.40	−1.30	.52
Caregiving → Infant cortisol I4	−.02	−.20	.01	.13	−.04	.01
Condition → Caregiving → Infant cortisol	−.04	.13	.05	.37	−.06	.15
*Model 2. Infant cortisol at the end of the visit (I5)*						
Condition → Infant Cortisol I5	.03	.10	.04	.43	−0.05	0.05
Condition → Caregiving	−.41	−.10	.46	.37	−1.32	.50
Caregiving → Infant cortisol I5	−.02	−.27	.01	.03	−.04	−.00
Condition → Caregiving → Infant cortisol	−.04	.13	.04	.33	−.04	.13
*Model 3. Infant crying*						
Condition → Infant crying	−.03	.06	.04	.54	−.06	.11
Condition → Caregiving	.37	−.09	.46	.42	−1.28	.53
Caregiving → Infant crying	−.05	−.46	.01	.00	−.07	−.03
Condition → Caregiving → Infant crying	.04	.10	.05	.36	−.05	.14

Condition is dummy coded with 0 = control condition and 1 = stressor condition. CI, confidence interval; LL, lower limit; SE, standard error; UL, upper limit.

#### Moderating role of maternal general postpartum distress

To assess the moderating role of maternal general postpartum distress, we tested four regression interaction models. All four models were not significant. Maternal general postpartum distress did not moderate the effect of maternal stressor on maternal caregiving behavior quality (*b* = −.11, *SE* = .19, *p* = .57) nor on infant response to maternal stress: cortisol at reunion (*b* = −.05, *SE* = .05, *p* = .35), cortisol at the end of the visit (*b* = −.04, *SE* = .04, *p* = .36), or crying (*b* = −.07, *SE* = .06, *p* = .23).

#### Maternal reactivity to stressors on caregiving behavior and infant cortisol and crying

To examine the associations between maternal reactivity to the acute stressor (i.e., cortisol and negative affect reactivity) and maternal caregiving behavior and infant response (i.e., cortisol at reunion, cortisol at the end of the visit, crying) regardless of condition, we ran six SEMs (see Table S2). The models revealed no significant associations between maternal reactivity to the stressor on caregiving behavior and infant cortisol and crying. Again, we found that higher maternal caregiving behavior quality was associated with lower infant cortisol at the end of the visit and less crying (not cortisol at reunion). Furthermore, we found some covariates to be significantly related to the outcome measures (see Table S2). Breastfeeding the infant before the manipulation was significantly related to lower infant cortisol at reunion, less infant crying, and lower maternal cortisol reactivity. The fit of the models can be found in Table S1.

### Sensitivity analyses

We also conducted sensitivity analyses for cortisol reactivity by testing the peaks M3 and M4 separately, subtracting each from the lowest baseline (M1 and M2). Notably, we found that reactivity, defined as the peak cortisol level at the end of the stressor (M4), was significantly related to higher infant cortisol levels at the end of the visit (I5; β = .28, *p* = .03). No significant associations were found with infant cortisol at reunion (I4; β = .21, *p* = .10) or infant crying (β = .15, *p* = .12). These findings were not replicated when reactivity was calculated using the peak from the start of the stressor (M3): infant cortisol at reunion (I4; β = .10, *p* = .38), the end of the visit (I5; β = .13, *p* = .26), and infant crying (β = .06, *p* = .53).

The sensitivity analysis assessing the association between maternal negative affect reactivity by either assessing negative affect at the start (VAS2) or the end of the manipulation (VAS3) on infant outcomes did not yield different results, as all analyses remained insignificant (all *p*'s > .09).

## Discussion

No evidence was found for mother‐infant stress contagion, with no main effects of maternal stressor exposure on infant cortisol or crying (hypothesis 1) and no mediation of this relation by caregiving behavior (hypothesis 2). Moreover, no evidence was found for a moderating effect of maternal postpartum distress on the effect of maternal stressor exposure on infant cortisol and crying (hypothesis 3), nor an association between maternal cortisol or negative affect reactivity (irrespective of condition) on infant cortisol, crying, and caregiving behavior (hypothesis 4). Secondary findings revealed that higher maternal caregiving behavior quality was associated with lower infant cortisol levels at the end of the visit and less crying. Moreover, sensitivity analysis revealed that when maternal cortisol reactivity was operationalized as the difference between cortisol levels at the end of the manipulation and the lowest baseline, higher maternal cortisol reactivity was associated with elevated infant cortisol levels at the end of the visit.

These results of no association between the acute maternal stressor and infant outcomes contrast those of the two previous studies showing that maternal stressor exposure increased infant sympathetic nervous system activation and avoidance behavior (Waters et al., 2014, 2017). As we utilized infant cortisol concentrations and crying behavior as outcome measures, it is possible that stress contagion happened but was reflected in different infant biomarkers (e.g., heart rate, skin conductance) or infant behaviors (e.g., motor or looking behavior). Our infants were also much younger compared to the infants from previous mother‐infant stress contagion studies by Waters et al. (2014, 2017, i.e., 8 weeks instead of 13 months). Infants might have been (too) young to pick up maternal stress, which is congruent with indications of infant's inability to process complex social information or feel other's emotions at this age (Grossmann & Johnson, 2007; Ruffman, Lorimer, & Scarf, 2017). However, sensitivity analyses revealed that when maternal cortisol reactivity was operationalized as the difference between cortisol levels at the end of the manipulation and the lowest baseline, higher maternal cortisol reactivity was associated with elevated infant cortisol levels at the end of the visit. This may suggest that contagion is more likely to occur when mothers have higher cortisol levels towards the end of the stressor and approaching the reunion with their infant, compared to the start of the stressor. However, this hypothesis requires further testing. Future research on mother‐infant stress contagion is advised to critically consider the timing of cortisol assessments and, more specifically, to measure maternal cortisol levels at least directly before the reunion with their infant.

Furthermore, no evidence was found that maternal stressor exposure caused changes in maternal caregiving quality. Although observational studies often show associations between maternal distress and caregiving quality (Booth et al., 2018), our study is one of the first to experimentally manipulate maternal stress. However, these non‐significant findings are consistent with prior research on natural disasters as stressors, which demonstrated that prenatal exposure to objective hardship during the Queensland flood was not associated with maternal caregiving quality (Austin et al., 2017; McLean et al., 2021). While maternal caregiving quality as measured by the Ainsworth scales is found to predict a range of child outcomes (Ainsworth & Bell, 1974), it is also moderately stable over time (Else‐Quest, Clark, & Tresch Owen, 2011; Huang, O'Brien Caughy, Lee, Miller, & Genevro, 2009; Madigan, Plamondon, Browne, & Jenkins, 2016). Hence, acute stressors may impact this relatively stable behavior less. Changes due to a stressor might be reflected in other, more subtle caregiving behaviors such as touch and gaze (Beebe et al., 2008, 2011; Granat, Gadassi, Gilboa‐Schechtman, & Feldman, 2017; Lotzin et al., 2015). Indeed, it has been found that mother and infant exposure to an experimental stressor caused no differences in maternal caregiving quality, but maternal vocalizations had a higher frequency and less prosody tone (Tronick, Mueller, DiCorcia, Hunter, & Snidman, 2021). Future research should investigate more fine‐grained maternal behaviors after acute stressors.

Secondary findings indicated that infants of mothers with higher caregiving quality displayed less crying after reunion and had lower cortisol concentrations at the end of the visit. As such, this study replicated earlier findings that higher maternal caregiving quality was related to quicker cortisol recovery in 3‐month‐old infants (Albers, Marianne Riksen‐Walraven, Sweep, & Weerth, 2008) and not the lack of an association found in 5‐week‐old infants (Jansen, Beijers, Riksen‐Walraven, & De Weerth, 2010). Mothers might also have been more gentle with the infant, resulting in lower cortisol levels in the infant (Brandes‐Aitken et al., 2022). Together, these findings indicate that while maternal behavior may be less effective in buffering cortisol recovery in 1‐month‐olds, this may change around 2–3 months of age, when caregiving behavior appears to facilitate infant cortisol recovery. Alternatively, heritability of HPA‐axis functioning may also play a role in this association (Zänkert et al., 2019).

An additional finding from this study is that infants of mothers with higher levels of general postpartum distress were observed to cry more during the mother‐infant interaction, replicating other studies that relied on maternal reports of infant crying (Lux, Müller, Reck, Liel, & Walper, 2023; Mohr et al., 2019). This finding may be attributed to the reduced energy levels of mothers experiencing heightened distress, which could impair their ability to comfort their infants and subsequently lead to increased episodes of infant crying (Kurth, Kennedy, Spichiger, Hösli, & Stutz, 2011). Notably, we did not observe a correlation between maternal caregiving and maternal postpartum distress levels (i.e., symptoms of depression, anxiety, and stress). In contrast to other studies on postpartum distress and caregiving, our sample consisted predominantly of low‐risk, healthy participants with self‐reported distress, which tends to yield more mixed results compared to studies assessing clinical samples or individuals with diagnosed mental health conditions (Booth et al., 2018). Lastly, we replicated previous studies (Altemus et al., 2001; Thanh, Lupien, & Walker, 2005) by inducing acute stress in postpartum women despite hormonal fluctuations that typically dampen cortisol reactivity (Bell, Erickson, & Carter, 2014; Prevost et al., 2014; Uvnas‐Moberg et al., 2020).

Next to several strengths, including the randomized controlled design and the observational measures of maternal caregiving behavior and infant crying, limitations should also be mentioned. The participants were mostly Dutch and had higher educational levels than average in the Netherlands, which limits our study's generalizability. Moreover, our sample size was smaller than expected due to the COVID‐19 pandemic. While the analyses showed acceptable model fits, simulations of our models showed that the models with maternal stressor condition as a predictor had just enough power. Finally, missingness of infant cortisol samples was high. While our model's imputation techniques avoided loss of power, future research should take care to collect more infant saliva for cortisol analyses. Moreover, as we found no effect of an acute stressor on maternal caregiving behavior, it would be interesting for future research to investigate if maternal caregiving behavior can act as a moderator within the association between maternal stress and infant outcomes, for example, by buffering infant cortisol and behavioral responses, which has been found in observational studies (e.g., Grant et al., 2009; Nazzari, Fearon, Rice, Molteni, & Frigerio, 2022).

## Conclusions

To our knowledge, this study is the first to examine the impact of an acute maternal stressor on infant cortisol and crying and maternal caregiving quality. While no evidence was found for stress contagion, secondary findings revealed that infants of mothers with higher caregiving quality cried less and had lower cortisol concentrations at the end of the visit. Experimental designs manipulating maternal stress contribute to crucial insights into mechanisms through which maternal postpartum stress affects infant development. This is particularly relevant given the high prevalence of maternal mental health problems during the postpartum period.

## Ethical considerations

This study was approved by the Ethics Committee of the Faculty of Social Sciences of the Radboud University (SW2017‐1303‐497), including three amendments (ECSW‐2019‐051, ECSW‐2019‐106, ECSW‐2020‐021). Participants gave informed consent.

**Key points**

- We did not find evidence for mother‐infant stress contagion at 8 weeks of age.
- Acute maternal stress did not lead to worse quality of maternal caregiving.
- Higher quality of maternal caregiving behavior was related to lower levels of infant crying and quicker cortisol recovery.

## Supporting information


**Table S1.** Fit indices for SEM.
**Table S2.** Results from the six mediation models.
**Figure S1.** Structural Equation models for hypothesis 1, 2, and 4.

## Data Availability

The research data are part of an ongoing longitudinal study but can be requested for scientific purposes from Carolina de Weerth (carolina.deweerth@radboudumc.nl).
