# Supplementary material for: Mother–infant stress contagion? Effects of an acute maternal stressor on maternal caregiving behavior and infant cortisol and crying
Source: J Child Psychol Psychiatry. 2025 Jan 21;66(7):1040–52. doi: 10.1111/jcpp.14119 (PMC12198937; doi:10.1111/jcpp.14119)
Supplement: Supplementary file 1 — Table S1. Fit indices for SEM. Table S2. Results from the six mediation models. Figure S1. Structural Equation models for hypothesis 1, 2, and 4. [file JCPP-66-1040-s001.docx]

**Supporting Information**

| **Table 1.** Fit indices for SEM of caregiving behavior quality mediating maternal stressor on infant cortisol and crying (hypothesis 1 and 2) and caregiving behavior quality mediating maternal cortisol or negative affect reactivity on infant cortisol and crying (hypothesis 4). | | | | |
| --- | --- | --- | --- | --- |
|  | Chi-square | CFI | RSMEA | SRMR |
| **Hypothesis 1 & 2** |  |  |  |  |
| Model 1 | .25 | .98 | .08 | .06 |
| Model 2 | .18 | .97 | .09 | .06 |
| Model 3 | .06 | .97 | .10 | .06 |
| **Hypothesis 4** |  |  |  |  |
| Model 1 | .31 | .95 | .07 | .07 |
| Model 2 | .27 | .95 | .07 | .07 |
| Model 3 | .29 | .98 | .04 | .06 |
| Model 4 | .51 | .98 | .04 | .06 |
| Model 5 | .28 | .94 | .07 | .07 |
| Model 6 | .36 | .99 | .04 | .06 |
| Note. CFI = Robust Comparative Fit Index, RMSEA = Robust Root Mean Square Error Of Approximation, SRMR = Standardized Root Mean Square Residual. The fit of all models was adequate, but not perfect, which is reasonable due to the predictor of experimental condition not adding to any of the three models. | | | | |

| **Table 2.** Results from the six mediation models (hypothesis 4) of maternal stress reactivity divided into 1) cortisol reactivity and 2) negative affect reactivity on infant stress response divided into 1) infant cortisol at reunion 2) infant cortisol at recovery 3) infant crying, mediated by caregiving behavior quality | | | | | | |
| --- | --- | --- | --- | --- | --- | --- |
| Model description | Estimate | Standardized Estimate (β) | SE |  | | |
|  |  |  |  | p-value | CI | |
|  |  |  |  |  | LL | UL |
| **Models with maternal cortisol reactivity as predictor** | | | | | | |
| **Model 1 Infant cortisol at reunion (I4)** |  |  |  |  |  |  |
| 1. Maternal cortisol reactivity 🡪 Infant Cortisol I4 | .01 | .15 | .01 | .24 | -.01 | .02 |
| Covariates within model |  |  |  |  |  |  |
| - *Infant cortisol baseline I3🡪 Infant Cortisol I4* | .09 | .09 | .13 | .48 | -.17 | .35 |
| - *Infant age* 🡪 *Infant Cortisol I4* | -.04 | -.22 | .03 | .16 | -.09 | .01 |
| - *Breastfeeding* 🡪*Infant Cortisol I4* | -.13 | -.33 | .05 | .01 | -.23 | -.04 |
| - *Babysitter* 🡪*Infant Cortisol I4* | -.07 | -.16 | .05 | .20 | -.17 | .04 |
| - *Breastfeeding* 🡪 *Maternal cortisol reactivity* | -1.49 | -.22 | .71 | .04 | -2.88 | -.10 |
| - *Food-intake* 🡪 *Maternal cortisol reactivity* | -1.26 | -.14 | .99 | .20 | -3.20 | .68 |
| - *Infant age* 🡪 *Caregiving* | -.17 | -.10 | .20 | .40 | -.56 | .22 |
| 2. Maternal cortisol reactivity 🡪 Caregiving | .01 | .02 | .07 | .84 | -.13 | .16 |
| 3. Caregiving 🡪 Infant cortisol I4 | -.02 | -.21 | .01 | .07 | -.04 | .00 |
| 4. Maternal cortisol reactivity 🡪 Caregiving 🡪 Infant cortisol I4 | .01 | .14 | .01 | .26 | -.01 | .02 |
| **Model 2. Infant cortisol at recovery (I5)** |  |  |  |  |  |  |
| Maternal cortisol reactivity 🡪 Infant Cortisol at recovery I5 | .01 | .18 | .01 | .14 | -.00 | .02 |
| Covariates within model |  |  |  |  |  |  |
| - *Infant cortisol baseline I3*🡪 *Infant Cortisol I5* | .07 | .08 | .13 | .59 | -.18 | .32 |
| - *Infant age* 🡪 *Infant Cortisol I5* | -.03 | -.18 | .02 | .14 | -.06 | .01 |
| - *Breastfeeding* 🡪*Infant Cortisol I5* | -.05 | -.14 | .04 | .25 | -.60 | .18 |
| - *Babysitter* 🡪*Infant Cortisol I5* | -.09 | -.25 | .04 | .04 | -.17 | -.00 |
| - *Breastfeeding* 🡪 *Maternal cortisol reactivity* | -1.49 | -.22 | .71 | .04 | -2.88 | -.10 |
| - *Food-intake* 🡪 *Maternal cortisol reactivity* | -1.26 | -.14 | .99 | .20 | -3.20 | .67 |
| - *Infant age* 🡪 *Caregiving* | -.21 | -.12 | .20 | .30 | -.60 | .18 |
| 2. Maternal cortisol reactivity 🡪 Caregiving | .01 | .02 | .07 | .87 | -.13 | .15 |
| 3. Caregiving 🡪 Infant cortisol I5 | -.02 | -.27 | .01 | .02 | -.04 | -.00 |
| 4. Maternal cortisol reactivity 🡪 Caregiving 🡪 Infant cortisol I5 | .01 | .17 | .01 | .16 | -.00 | .02 |
| **Model 3 Infant crying** |  |  |  |  |  |  |
| Maternal cortisol reactivity 🡪 Infant crying | .01 | .13 | .01 | .14 | -.00 | .02 |
| Covariates within model |  |  |  |  |  |  |
| - *Infant age* 🡪 *Infant crying* | .04 | .19 | .02 | .03 | .00 | .07 |
| - *Breastfeeding* 🡪*Infant crying* | -.12 | -.26 | .04 | .00 | -.21 | -.04 |
| - *Babysitter* 🡪*Infant crying* | -.09 | -.19 | .04 | .02 | -.17 | -.01 |
| - *Breastfeeding* 🡪 *Maternal cortisol reactivity* | -1.50 | -.22 | .71 | .04 | -2.89 | -.11 |
| - *Food-intake* 🡪 *Maternal cortisol reactivity* | -1.27 | -.14 | .99 | .20 | -3.20 | .67 |
| - *Infant age* 🡪 *Caregiving* | -.17 | -.10 | .20 | .40 | -.56 | .22 |
| 2. Maternal cortisol reactivity 🡪 Caregiving | .01 | .02 | .07 | .88 | -.13 | .15 |
| 3. Caregiving 🡪 Infant crying | -.05 | -.46 | .02 | .00 | -.07 | -.03 |
| 4. Maternal cortisol reactivity 🡪 Caregiving 🡪 Infant crying | .01 | .13 | .01 | .22 | -.01 | .02 |
| **Models with Maternal Negative Affect reactivity as predictor** | | | | | | |
| Model description | Estimate | Standardized Estimate (β) | SE |  | | |
|  |  |  |  | p-value | CI | |
|  |  |  |  |  | LL | UL |
| **Model 4 Infant cortisol at reunion (I4)** |  |  |  |  |  |  |
| 1. Maternal Negative Affect reactivity 🡪 Infant Cortisol I4 | .02 | .08 | .03 | .59 | -.04 | .07 |
| Covariates within model |  |  |  |  |  |  |
| *Infant cortisol baseline I3🡪 Infant Cortisol I4* | .06 | .06 | .14 | .65 | -.21 | .33 |
| *Infant age* 🡪 *Infant Cortisol I4* | -.04 | -.23 | .03 | .14 | -.09 | .01 |
| *Breastfeeding* 🡪*Infant Cortisol I4* | -.14 | -.35 | .05 | .01 | -.24 | -.04 |
| *Babysitter* 🡪*Infant Cortisol I4* | -.06 | -.14 | .05 | .27 | -.16 | .04 |
| *Breastfeeding* 🡪 *Maternal Negative affect reactivity* | .18 | .09 | .22 | .42 | -.25 | .59 |
| *Food-intake* 🡪 *Maternal Negative affect reactivity* | .03 | .01 | .30 | .92 | -.56 | .62 |
| *Infant age* 🡪 *Caregiving* | -.18 | -.10 | .19 | .36 | -.56 | .20 |
| 2. Maternal Negative affect reactivity 🡪 Caregiving | .03 | .01 | .24 | .91 | -.44 | .49 |
| 3. Caregiving 🡪 Infant cortisol I4 | -.02 | -.21 | .01 | .07 | -.04 | .00 |
| 4. Maternal Negative affect reactivity 🡪 Caregiving 🡪 Infant cortisol I4 | .01 | .07 | .03 | .61 | -.04 | .07 |
| **Model 5 Infant Cortisol at Recovery (I5)** |  |  |  |  |  |  |
| 1. Maternal Negative affect reactivity 🡪 Infant Cortisol I5 | .02 | .14 | .02 | .24 | -.02 | .06 |
| Covariates within model |  |  |  |  |  |  |
| *Infant cortisol baseline I3🡪 Infant Cortisol I5* | .03 | .04 | .13 | .81 | -.22 | .29 |
| *Infant age* 🡪 *Infant Cortisol I5* | -.03 | -.22 | .02 | .07 | -.06 | .00 |
| *Breastfeeding* 🡪*Infant Cortisol I5* | -.67 | -.19 | .04 | .12 | -.15 | .02 |
| *Babysitter* 🡪*Infant Cortisol I5* | -.08 | -.23 | .04 | .06 | -.16 | .00 |
| *Breastfeeding* 🡪 *Maternal Negative affect reactivity* | .17 | .08 | .22 | .43 | -.25 | .60 |
| *Food-intake* 🡪 *Maternal Negative affect reactivity* | .03 | .01 | .30 | .92 | -.56 | .61 |
| *Infant age* 🡪 *Caregiving* | -.21 | -.12 | .20 | .28 | -.59 | .17 |
| 2. Maternal Negative affect reactivity 🡪 Caregiving | -.01 | -.00 | .24 | .99 | -.47 | .46 |
| 3. Caregiving 🡪 Infant cortisol I5 | -.02 | -.12 | .20 | .03 | -.04 | -.00 |
| 4. Maternal Negative affect reactivity 🡪 Caregiving 🡪 Infant cortisol I5 | .02 | .14 | .02 | .25 | -.02 | .06 |
| **Model 6 Infant crying** |  |  |  |  |  |  |
| Maternal Negative affect reactivity 🡪 Infant crying | -.01 | -.03 | .02 | .71 | -.05 | .03 |
| Covariates within model |  |  |  |  |  |  |
| *Infant age* 🡪 *Infant crying* | .03 | .17 | .02 | .06 | -.00 | .07 |
| *Breastfeeding* 🡪*Infant crying* | -.13 | -.28 | .04 | .00 | -.22 | -.05 |
| *Babysitter* 🡪*Infant crying* | -.09 | -.19 | .04 | .03 | -.17 | -.01 |
| *Breastfeeding* 🡪 *Maternal Negative affect reactivity* | .18 | .09 | .22 | .42 | -.25 | .60 |
| *Food-intake* 🡪 *Maternal Negative affect reactivity* | .03 | .01 | .30 | .91 | -.55 | .62 |
| *Infant age* 🡪 *Caregiving* | -.17 | -.10 | .19 | .37 | -.55 | .21 |
| 2. Maternal Negative affect reactivity 🡪 Caregiving | .03 | .02 | .24 | .89 | -.43 | .50 |
| 3. Caregiving 🡪 Infant crying | -.05 | -.46 | .01 | .00 | -.07 | -.03 |
| 4. Maternal Negative affect reactivity 🡪 Caregiving 🡪 Infant crying | -.01 | -.04 | .02 | .69 | -.06 | .04 |
| Note. SE= Standard Error, CI=Confidence interval, LL= Lower limit, UL= Upper limit. Breastfeeding before manipulation is dummy coded as 0= not breastfed, 1 = breastfed. Babysitter is dummy coded as 0 = brought by the mother, 1= research assistant | | | | | | |

**Figure 1** Structural Equation models for hypothesis 1 and 2 (model A) and hypothesis 4 (model B) including all covariates.


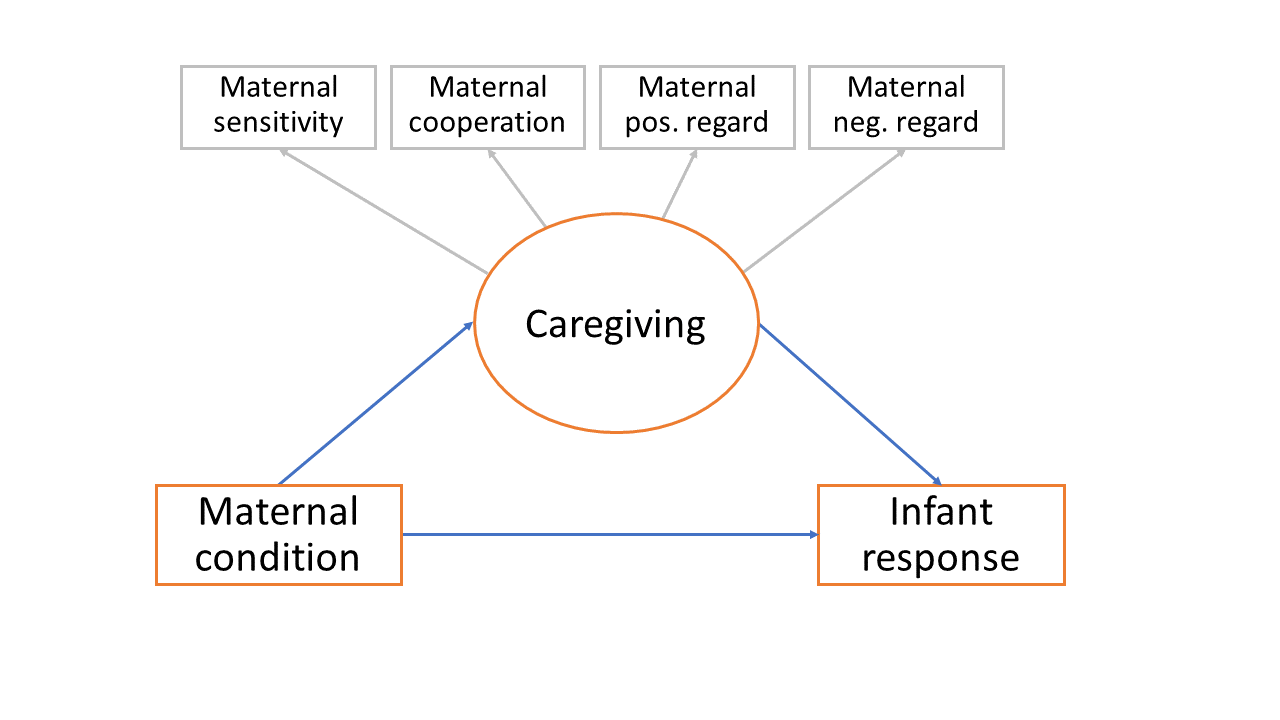


**A.**


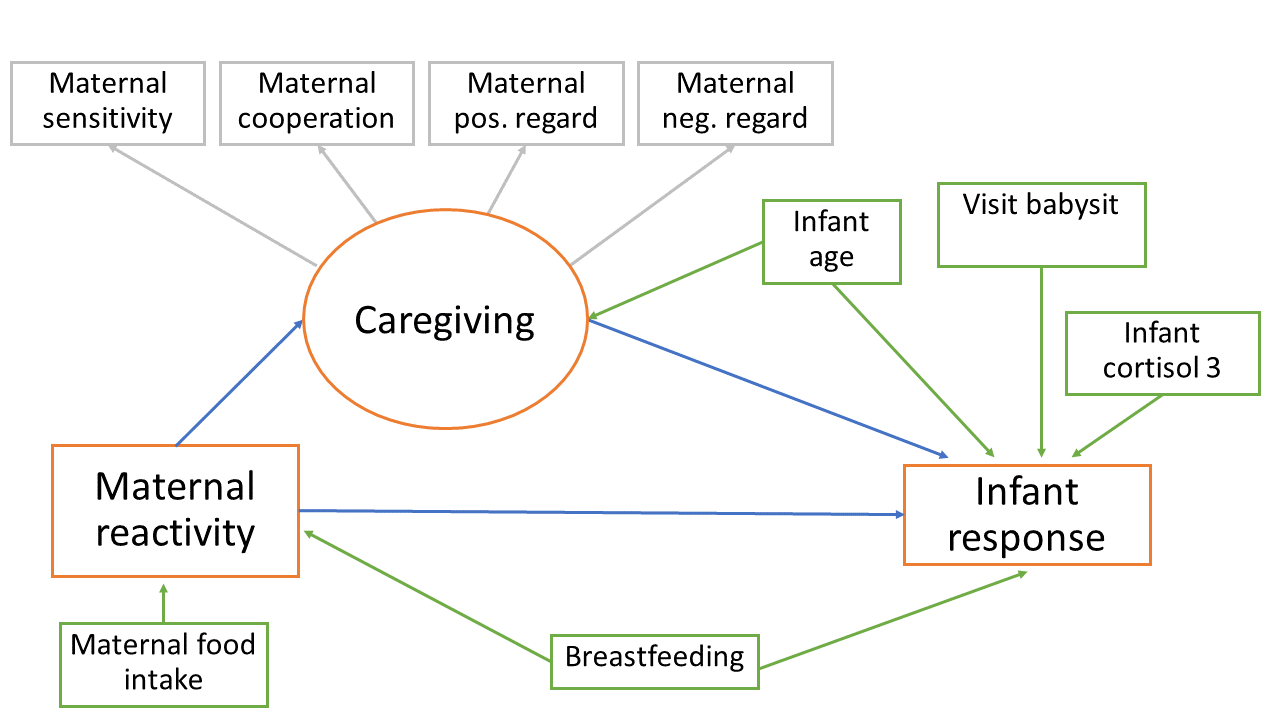
Note.

Note. Infant response reflects infant cortisol 4 (I4), infant cortisol 5 (I5) and infant crying. Boxes in orange reflect the study variables, with the blues lines reflecting the mediation model. Caregiving was a latent score consisting of the latent factors in grey. Boxes in green reflect the covariates. Maternal reactivity reflects cortisol and negative affect reactivity. For model B infant cortisol 3 (reflecting baseline cortisol) was not added for the model with infant crying, only for the models testing I4 and I5.

**B.**
